# Under the radar: a cross-sectional study of the challenge of identifying at-risk alcohol consumption in the general practice setting

**DOI:** 10.1186/1471-2296-15-74

**Published:** 2014-04-28

**Authors:** Christine Paul, Sze Lin Yoong, Rob Sanson-Fisher, Mariko Carey, Grant Russell, Meredith Makeham

**Affiliations:** 1 Health Behaviour Research Group, Priority Research Centre for Health Behaviour and Hunter Medical Research Institute, The University of Newcastle, Callaghan, NSW, Australia; 2 School of Public Health and Community Medicine, University of Sydney, Sydney, NSW, Australia; 3 Southern Academic Primary Care Research Unit, School of Primary Health Care, Monash University, Clayton, VIC, Australia

**Keywords:** General practice, Alcohol, Detection, Accuracy, Sensitivity

## Abstract

**Background:**

Primary care providers are an important source of information regarding appropriate alcohol consumption. As early presentation to a provider for alcohol-related concerns is unlikely, it is important that providers are able to identify at-risk patients in order to provide appropriate advice. This study aimed to report the sensitivity, specificity, positive predictive value and negative predictive value of General Practitioner (GP) assessment of alcohol consumption compared to patient self-report, and explore characteristics associated with GP non-detection of at-risk status.

**Method:**

GP practices were selected from metropolitan and regional locations in Australia. Eligible patients were adults presenting for general practice care who were able to understand English and provide informed consent. Patients completed a modified AUDIT-C by touchscreen computer as part of an omnibus health survey while waiting for their appointment. GPs completed a checklist for each patient, including whether the patient met current Australian guidelines for at-risk alcohol consumption. Patient self-report and GP assessments were compared for each patient.

**Results:**

GPs completed the checklist for 1720 patients, yielding 1565 comparisons regarding alcohol consumption. The sensitivity of GPs’ detection of at-risk alcohol consumption was 26.5%, with specificity of 96.1%. Higher patient education was associated with GP non-detection of at-risk status.

**Conclusions:**

GP awareness of which patients might benefit from advice regarding at-risk alcohol consumption appears low. Given the complexities associated with establishing whether alcohol consumption is ‘at-risk’, computer-based approaches to routine screening of patients are worthy of exploration as a method for prompting the provision of advice in primary care.

## Background

Alcohol-related harm is estimated to contribute 3.2% of the total burden of disease and injury in Australia [1], with concerning levels of hazardous drinking evident in a number of countries [2,3]. Alcohol is second only to tobacco as a preventable cause of drug-related death and hospitalisation [4]. Alcohol is a causal factor in about 60 types of diseases, whether resulting from short-term episodes of intoxication or from long-term, chronic use [1,5]. In 2004–05, the total social cost in Australia of alcohol-related social problems was estimated to be $15.3 billion; of which most was due to tangible costs such as lost productivity, health, road accidents and crime [6]. Estimates of the proportion of adults in Australia who consume alcohol above the recommended level, or who are ‘at-risk’ due to their alcohol consumption range upwards from 7% [7], and estimates of the prevalence of alcohol use disorders are 5% or higher in a number of European countries [3].

Internationally, guidelines regarding the safe consumption of alcohol have successively recommended lower levels of consumption [8-10]. In Australia for example, the 2009 National Health and Medical Research Council (NHMRC) guidelines advised both men and women to drink no more than two standard drinks per day [4]. The previous guidelines set out four drinks for men and two drinks for women per day, on average [11]. Not surprisingly, a recent Australian survey found less than 5% of respondents were able to accurately name low-risk levels for short- and long-term drinking [12].

In the absence of concerted public education campaigns regarding the level of drinking which may place health at risk, primary care providers are likely to be perceived by the community as an important source of information regarding appropriate alcohol consumption. According to the Patient Experience Survey, 81% of people aged 15years and over had seen a general practitioner (GP) in the prior 12 months [13]. GPs play an important role in monitoring an individual’s health and managing their health conditions, with patients expecting to receive preventive health advice from their GP [14]. Brief advice from doctors has been shown to be effective in changing a range of health risk behaviours such as alcohol misuse, tobacco smoking and physical activity [15]. However, in the absence of systematic approaches to detect alcohol consumption in patients, it may be very challenging for general practitioners to identify and assist those who might benefit from advice about alcohol consumption.

While there are no current data on rates of detection of at-risk alcohol consumption in general practice, a small group of studies suggest detection rates may be of concern. In 1986 Reid et al. [16] found general practitioners correctly identified 27.5% of patients who were classified as “high risk” drinkers according to self-reported consumption. Two studies have explored GPs’ ability to detect alcohol-related problems such as dependence or abuse [17,18]. The more recent study found that when GPs were asked whether a patient had alcohol dependence or abuse, sensitivity was 50.4% when compared to a positive diagnosis on clinical screening instruments (CAGE and SMAST) and 74.4% compared to a diagnosis of alcohol dependence or abuse using the SCAN research interview [17]. However, only 34.4% of physicians surveyed in 1999 reported regularly screening for alcohol abuse or dependence [19], and more than a quarter of GPs surveyed in 2003 were unaware of the safe drinking levels for men and women [20]. As current recommendations regarding at-risk alcohol consumption are substantially lower than levels previously associated with dependence or abuse, at-risk consumption may now be more prevalent than when these earlier studies were conducted. If detection levels are low, a sizeable proportion of at-risk patients may go unnoticed. Therefore, it is timely to examine rates of detection of at-risk alcohol consumption in the general practice setting and also to explore whether detection rates vary by patient characteristics.

The primary method of screening for alcohol problems in general practice continues to be by self-report instruments [21]. Retrospective Diaries and Quantity-Frequency Questions have been used as a method for self-report of alcohol consumption, with diaries the more accurate method [22]. As the length of diary measures can be prohibitive for routine screening, one of the most commonly used screening instruments is the World Health Organisation’s AUDIT (the Alcohol Use Disorder Identification test) which has a Test–retest reliability of 0.86 in primary care [23]. Other screening tools such as the CAGE, SMAST and SCAN are more lengthy than the three-item AUDIT-C and more suited to screening for alcohol dependence rather than the lower levels of use recommended in current guidelines for alcohol consumption. The full AUDIT and three item AUDIT-C questionnaires are effective in screening for harmful drinking levels (sensitivity 51-97%, specificity 78-96%) [24-26], and can be used to assess alcohol consumption against current Australian national guidelines with very minor modification, i.e. to specify 4 rather than 6 drinks as representing ‘at-risk’ alcohol consumption. Research supports the use of formal self-report screening instruments as opposed to clinical indicators such as biomarkers of heavy drinking [26].

A comparison of GPs’ assessments of whether their patients meet current guidelines for at-risk alcohol consumption with patients’ self-reported alcohol consumption using items from the AUDIT-C is likely to provide an indication of the degree to which GPs are aware of which patients might benefit from advice regarding alcohol consumption. This would provide an indication of whether the use of screening tools is required in order to provide a mechanism for preventive educating and advice for patients regarding alcohol consumption.

The study aimed to:

1. Report the sensitivity, specificity, positive predictive value and negative predictive value of GP-assessment compared to patient self-report regarding whether alcohol consumption was at-risk according to national guidelines.

2. Identify whether socio-demographic characteristics such as age group, gender, education, ethnicity, having a health care card, having private health insurance and frequency of GP visits were associated with a GP not detecting patients’ self-reported at-risk alcohol consumption.

## Method

The data presented here were collected as part of a larger study exploring chronic-disease related issues in general practice, as described in detail elsewhere [27]. The study was approved the by the Human Research Ethics Committees of the University of Newcastle, Monash University and University of New South Wales.

### Recruitment of general practices and practitioners

GP practices were selected from the Melbourne, Newcastle and Sydney regions and generated from the Medical Directory Australia database and the “yellow pages”, an online telephone directory. General practices were eligible if at least two full time GPs consented to participate. Postcodes in each area were generated and practices within a randomly selected postcode approached. A package containing an invitation letter, information statement and consent form was sent out to GPs and practice managers. Two follow up phone calls were made to the practice and additional information sent out if requested.

### Recruitment of patients

#### Eligibility criteria

Eligible patients were presenting for general practice care; aged 18 or older; able to understand English; and able to provide informed consent.

#### Recruitment of patients

Consecutive eligible participants were approached in the waiting room of the general practice and invited to participate in an omnibus survey regarding testing the acceptability of touch screen computers for assessing health risk factors among general practice patients. The information statement described the main categories of survey items (eg cancer risk behaviours) but did not specifically mention alcohol. Informed consent was sought from all participants. Estimated age and gender were recorded for potentially eligible non-participants.

#### Data collection

Patients completed the survey by touchscreen computer in the waiting room of the general practice prior to their appointment. For a randomly selected subsample of participants, GPs were asked to complete a hard copy checklist regarding whether the patient had each of six health risks and whether they had completed appropriate health screening. The research assistant handed the checklist to the GP according to the GP’s pre-specified preference for completing the checklist during or after the consultation. No GP chose to complete the checklists prior to the consultation. Most chose to complete the checklists at the time of each consultation, or at the end of the day.

### Measures

#### Patient survey

Age, gender, education, private health insurance, number of GP visits in last 12 months and whether or not the patient had a commonwealth health care concession card were recorded. Items on previous medical history, current health status and recent care were recorded for depression, blood pressure, cholesterol, heart disease, diabetes, cancer, stroke and chronic pain by all participants. The survey items relating to alcohol consumption were the modified version of AUDIT-C items:

“*How often do you usually have a drink containing alcohol?”*

(Never, Monthly or less, 2–4 times per month (once a week or once every 2weeks), 2–3 times per week, 4 or more times per week);

“On a typical day that you have an alcoholic drink, how many STANDARD drinks do you usually have?” (Note: One middy/100 mls of wine = 1 standard drink; One schooner/375 ml premixed can = 1.5 standard drinks; One bottle of wine = 7 standard drinks)”.

A graphic depicting standard drinks was displayed;

“*How often do you have 4 or more drinks on one occasion?”*

(Never, Less than monthly, Monthly Weekly, Daily or almost daily).

#### GP checklist

The one-page checklist asked:

“Does the patient have any of the following health risks? Current cigarette smoker, overweight, obese, clinical depression, risky alcohol consumption, inadequate exercise”.

(Yes, No, Unsure for each risk). Each checklist had an attached cover sheet defining each health risk. For risky alcohol consumption it stated:

“A patient is considered at risk if their alcohol consumption levels are more than that recommended by the 2009 NHMRC Guidelines for reducing risk of alcohol-related harm over a lifetime (≥2 standard drinks daily or almost daily) OR if they usually have four or more standard drinks on one occasion (weekly, daily or almost daily)”.

### Statistical analyses

All data analyses were conducted using STATA 11.0. GPs and patient reported information were matched using a unique ID provided to patients. Patient responses regarding alcohol consumption were dichotomised into ‘at-risk’ and ‘not at-risk (see below for definition). Similarly, GP responses were also grouped into the same two categories depending on whether GPs indicated ‘Yes’ for at-risk drinking in their patients. Where the GP indicated ‘not sure’ those results were excluded from the primary analysis. A sensitivity analysis was conducted with the ‘not sure’ responses re-categorised as a ‘no’. The percentage and 95% confidence intervals (CIs) of at-risk drinking as reported by patient and GPs were reported. The sensitivity, specificity, positive predictive value and negative predictive values and corresponding 95% CIs were calculated. Sensitivity was calculated as the proportion of those patients at risk for whom the GP indicated ‘yes’ the patient had risky alcohol consumption. Specificity was calculated as the proportion of those patients not at risk for whom the GP indicated ‘no’ the patient did not have risky alcohol consumption. All 95% CIs were controlled for clustering using the svyset command.

In order to identify patient characteristics associated with GP identification of at-risk alcohol consumption, multiple logistic regression analysis adjusted for clustering within practices was conducted. Only patients who were defined as having an at-risk alcohol intake were included in this analysis (with unsure coded as ‘no’). A dichotomous variable was generated based on a) Patient-GP agreement regarding at-risk alcohol consumption and b) Patient-GP disagreement regarding at-risk alcohol consumption. Univariate analysis was first performed with all patient and GP characteristic variables. Variables examined in the initial model included: sex (Male/Female), has type 2 diabetes (Yes/No), high blood pressure (Yes/No), high cholesterol (Yes/No), history of heart disease (Yes/No), age (18–29, 30–44, 45–64, ≥65), has private health insurance (Yes/No), number of times seen GP in last 1months (0–3, 4–6, 7–10, ≥10), Ethnicity (Caucasian/Non-Caucasian). Variables with a p-value of >0.1 on the Adjusted Wald test were included in the final model. Odds ratios with 95% CIs and p-values of univariate and adjusted models are presented.

#### Calculation of at-risk status

At-risk alcohol consumption was defined as more than 2 drinks 2–3 times a week or four or more times a week and/or >4 drinks weekly, daily or almost daily).

## Results

### Sample

Of the 81 GPs within consenting practices; 53 consented to participate and 51(63%) completed at least one checklist. The GP sample was similar to that of all Australian GPs [28] in that 63% were male, 57% were aged 50 years or older, 65% had been in general practice for more than 20 years and 69% worked full-time.

#### Patients

A total of 5671 patients were approached to participate in the larger study, of which 4079 agreed to participate (86% of those eligible). Of the patients presenting for general practice care; 17% were ineligible (3% non-English speaking; 42.7% younger than 18, 11% unable to complete survey, 2.8% were presenting for care to allied health practitioners and 40% for other reasons). Comparison of the estimated age and gender of non-consenting patients with consenting patients found no significant differences between the two groups. Of the 1720 consecutive patients selected for checklist completion, 1607 completed the alcohol items.

#### GPs

GPs completed the checklist for 1720 patients. No significant differences in age or gender were identified regarding the patients for whom GPs did or did not complete the checklist. From the 1720 GP checklists, 155 were excluded from the sample (113 patients did not complete the alcohol items, 41 GP checklists did not have the alcohol item completed, 1 record was ineligible), leaving 1565 available comparisons.

#### Accuracy (Sensitivity, specificity, positive predictive value & negative predictive value)

Initial analysis of sensitivity and specificity excluded those where the GP provided an ‘unsure’ rating (n = 216), leaving a sample of 1349 comparisons. Table 1 shows the level of agreement between GP assessment of alcohol consumption and patient self-report. The sensitivity of GPs’ detection of at-risk alcohol consumption was 26% (95% CI = 19, 35), with specificity of 96% (93, 98). Positive predictive value of GPs in detecting at-risk alcohol consumption was 63% (58, 65), with negative predictive value at 84% (80, 87).

**Table 1:** **Agreement between GP assessment of at**-**risk alcohol consumption and patient self**-**report** (**GP unsure responses excluded**, **n** = **1349**)

**GP**	**Patient reported**				
	At-risk*		**Not at-risk**		**Total**
	**n (%)**	**95% CI**	**n (%)**	**95% CI**	
At risk	71 (26)	[19, 35]	42 (3.9)	[2.2, 6.7]	113 (8.4) [5.6, 12]
Not at-risk	197 (74)	[65, 81]	1039 (96)	[93, 98]	1236 (92) [88, 94]
	268 (20)	[17,23]	1081 (80)	[77,87]	1349

*at-risk defined as more than 2 drinks 2–3 times a week or four or more times a week and/or >4 drinks weekly, daily or almost daily).

If the ‘unsure’ ratings are considered to be a ‘no’ (on the assumption that being unsure is less likely to result in a GP exploring alcohol consumption), the level of sensitivity of GPs’ detection of at-risk alcohol consumption was 22% (95% CI = 15, 31), with specificity of 97% (94, 98). Positive predictive value of GPs in detecting at-risk alcohol consumption was 63% (58, 65), with negative predictive value of 83% (79, 87) (see Table 2).

**Table 2:** Agreement between GP assessment of at-risk alcohol consumption and patient self-report (with GP unsure coded as no, n = 1,565)

**GP**	**Patient reported**				
	At-risk*		**Not at-risk**		**Total**
	**n (%)**	**95% CI**	**n (%)**	**95% CI**	
At risk	71 (22)	[15,31]	42 (3.4)	[2.0,5.8]	113 (7.2) [4.8, 11]
Not at-risk	248 (78)	[69, 85]	1204 (97)	[94, 98]	1452 (93) [89, 95]
	319 (20)	[17,23]	1246 (80)	[77,83]	1,565

*at-risk defined as more than 2 drinks 2–3 times a week or four or more times a week and/or >4 drinks weekly, daily or almost daily).

### Patient characteristics associated with GP non-detection of self-reported at-risk status

For the group who were at-risk based on self-reported consumption (n = 268, which includes the ‘don’t knows’ coded as missing), logistic regression analysis was used to explore whether various characteristics were associated with the GP reporting that the patient was not at risk. Non-detection by the GP was associated with having tertiary rather than Higher School Certificate or lesser education (2.8, 95% CI = 1.1, 6.9) (see Table 3).

**Table 3:** Demographic characteristics associated with GP non-detection of self-reported at-risk* status (n = 268)

**Variables**	**Univariate analysis**			**Final model (*n =*** **242)**		
	**Crude odds ratio**	**95% CI**	**p-value**	**Odds ratio**	**95% CI**	**p-value**
**Age (*n =*** **268)**			0.1649			
18 - 24	1					
25 - 44	1.7	[0.5, 5.3]				
45 - 64	1	[0.3, 3.0]				
65+	1.6	[0.5, 4.9]				
**Sex (*n =*** **268)**			0.115			
Male	1					
Female	1.8	[0.8, 3.9]				
**Ethnicity (*n =*** **268)**			0.0411			
Non-Caucasian	1			1		
Caucasian	2.1	[1.0, 4.4]		1.8	[0.9, 3.8]	0.064
**Education (*n =*** **242)**^**a**^			0.0415			
HSC and below	1			1		
TAFE/diploma	2	[0.8, 5.1]		1.7	[0.7, 4.0]	0.204
Tertiary and above	3.4	[1.4, 8.0)		2.8	[1.1, 6.9]	0.033*
**Commonwealth card (*n =*** **253)**^**a**^						
No	1					
Yes	0.8	[0.4, 1.3]	0.303			
**Private health (*n =*** **253)**^**a**^				1		
No	1			1.8	[0.9, 3.8]	0.098
Yes	2.2	[1.1, 4.3]	0.266			
**Number of times seen GP (*n =*** **258)**			0.429			
0-3	1					
4-6	0.9	[0.4, 2.2]				
7-10	0.6	[0.3, 1.4]				
10+	0.5	[0.2, 1.4]				

*at-risk defined as more than 2 drinks 2–3 times a week or four or more times a week and/or >4 drinks weekly, daily or almost daily).

^**a**^Number less than total due to incomplete participant surveys.

## Discussion

The study findings are among the few to indicate the degree to which GPs identify whether their patients consume alcohol at a level that would be considered at risk of negative health consequences. Given the number of challenges associated with making such identifications in the study context, it is not surprising that only a minority of patients who reported at-risk levels of consumption were identified by their GP.

The low sensitivity (26.5% or 22.3%) and moderate positive predictive value found in the sample suggests that GPs either under-estimate their patients’ alcohol consumption, or misinterpret the current guidelines regarding at-risk alcohol consumption. The identified levels of sensitivity for at-risk alcohol consumption are lower than those reported for other health behaviours such as 56% for smoking [29]. The finding that in 13.7% of cases, the GP was not able to estimate whether the patient’s consumption of alcohol was of an at-risk level, combined with a poor level of sensitivity, suggests that alcohol consumption is not commonly discussed in general practice.

The data must be interpreted in light of the likelihood that patients may under-report alcohol consumption to their health care provider. Even allowing for such likelihood, the implication of very poor sensitivity is that without some alternative strategy, many of those who may benefit are unlikely to receive advice about alcohol consumption from their general practitioner. Given patients’ reliance on GPs to provide preventive health advice [14], this is likely to mean that many attendees to general practice may believe that their current alcohol consumption does not place their health at risk. This is particularly likely for those whose consumption exceeds the current guidelines by a small to moderate margin. It is this group which may be most able to adapt their level of drinking in response to brief advice. While there has been some public education in Australia regarding at-risk alcohol consumption, much of this has related to when it is safe to drive a vehicle. Public education thus far is unlikely to have been sufficient to ensure the community is fully aware of how to minimise the future risks associated alcohol consumption.

Also of note is the finding that those with higher education were at higher odds than those with lower education to fail to be identified as at-risk by their GP. While this may not be surprising, it further supports the need for the implementation of screening tools to ensure that detection and hence advice is provided equitably.

The challenges associated with assessing alcohol consumption in the busy general practice setting include the complexity of understanding the size of a standard drink, the tendency to under-report consumption, and the calculations associated with comparison of behaviour with guidelines. The use of tailored graphics and algorithmic software can reduce some of these complexities and assist patients to identify their level of alcohol consumption prior to consulting the GP. The successful electronic waiting room survey approach used in this study suggests that such an approach could be used to: i) facilitate brief advice from GP during the consultation via computer-generated prompts; ii) provide automated feedback direct to the patient before the consultation or iii) prompt a referral or subsequent consultation to address alcohol consumption along with other preventive health issues. While the effectiveness of such approaches for reducing alcohol consumption in general practice is yet to be robustly demonstrated, the results of this study suggest the need for further exploration. The public health benefit of bringing alcohol consumption to a level at or below the guideline-recommended level on a population level is likely to be substantial, given the range of diseases associated with alcohol consumption [1,5]. While there may be an opportunity cost for busy general practitioners in focussing on a level of alcohol consumption which may seem relatively low, as with other preventive interventions, the benefits of population-level change outweigh the costs [3,30].

### Study limitations

A number of limitations must be considered when interpreting the findings. First, relatively recent changes in the national guidelines on alcohol consumption may have limited the opportunity for GPs to consider current recommendations and explore alcohol-related issues with patients. Therefore, it is possible that higher levels of sensitivity would have been achieved had the study been conducted some years later. Second, an alcohol diary is considered to provide the best quality self-reported consumption data, but was not possible in the context of the omnibus survey in which the reported items were embedded. It is difficult to estimate the likely effects of under-reporting on the study findings given that this factor is likely to have impacted on both the result of the patient survey, and the prior information each GP would have gained from his patient about their alcohol consumption. It is possible that these two effects may have effectively cancelled each other out. Third, the involvement of 51 GPs from 12 practices may not provide a highly representative sample of all GPs. However, given the nature of the larger study, if bias has occurred it is likely to be towards including those practices and GPs who have a greater interest in the diagnosis of chronic disease. Therefore overall, the study is likely to provide a conservative estimate of the degree to which at-risk alcohol consumption is being diagnosed.

## Conclusions

In the absence of implementation of screening tools, the potential benefits of advice regarding at-risk alcohol consumption are unlikely to be realised in the general practice setting. This is particularly so for those with higher education. There is likely to be population benefit from a consistent approach to the assessment of alcohol consumption in primary care settings. Given the complexities and challenges associated with detecting whether alcohol consumption should be considered as ‘at-risk’ computer-based approaches to this challenge are worthy of exploration.

## Abbreviations

GP: General practitioner; CAGE: Cut down, annoyed, guilty, and eye-opener; SMAST: Short Michigan Alcohol Screening Test; CI: Confidence interval.

## Competing interests

All authors declare that they have no competing interests.

## Authors’ contributions

CP, SY, RSF, MC and MM contributed to the study concept and design. SY, GR and MM collected the data. SY and CP analysed the data. CP drafted the manuscript. All authors read, revised and approved of the final manuscript.

## Pre-publication history

The pre-publication history for this paper can be accessed here:

http://www.biomedcentral.com/1471-2296/15/74/prepub
